# Effects of intramuscular fat on meat quality and its regulation mechanism in Tan sheep

**DOI:** 10.3389/fnut.2022.908355

**Published:** 2022-07-28

**Authors:** Xueying Zhang, Chongyang Liu, Yuanyuan Kong, Fadi Li, Xiangpeng Yue

**Affiliations:** State Key Laboratory of Grassland Agro-Ecosystems, Key Laboratory of Grassland Livestock Industry Innovation, Ministry of Agriculture and Rural Affairs, Engineering Research Center of Grassland Industry, Ministry of Education, College of Pastoral Agriculture Science and Technology, Lanzhou University, Lanzhou, China

**Keywords:** intramuscular fat, meat quality, metabolomics, genetic regulation, Tan sheep

## Abstract

Intramuscular fat (IMF) contributes importantly to various aspects of meat quality, and genetic regulation is an effective pathway to improve IMF deposition in sheep. In this study, we systematically explored the effect of IMF content on meat quality in Tan sheep and investigated the regulatory mechanism of flavor precursors metabolism and IMF deposition. The results revealed that IMF significantly affected meat color, total muscle fiber numbers, and muscle fiber types in Tan sheep. Widely-targeted metabolomic analysis showed that amino acids were the main differential flavor precursors between lambs with different IMF content. Importantly, the comparison of fatty acid profiles revealed that saturated fatty acids and monounsaturated fatty acids are beneficial for IMF deposition. Furthermore, integrated analysis between metabolome and transcriptome indicated that *MME* is a key gene resulting in the reduction of amino acids in lambs with high IMF content; and the joint analysis between fatty acid profiles and transcript profiles showed that *ADIPOQ*, *FABP4*, *PLIN1*, *PPARGC1A*, *SLC2A1* accelerated IMF deposition through positive regulation of saturated fatty acids and monounsaturated fatty acids metabolism. These results revealed key changes in meat quality affected by IMF content and the corresponding genetic mechanism, which may provide a new insight for understanding the IMF differential deposition and for improving meat quality in Tan sheep.

## Introduction

With the increasing awareness of consumers of the origin of meat products and their associated quality, producing high-quality mutton has brought about new challenges for the meat industry (1). Meat quality can be comprehensively assessed by sensory quality, nutritional quality, processing quality, and safe quality, among which sensory quality is an essential indicator that determines consumer acceptance for meat products (2, 3). Therefore, it is of great significance to improve the sensory quality of mutton to meet the increasing needs of consumers. Meat sensory properties mainly comprise meat color, tenderness, juiciness, and flavor, which influence consumer choice at the point of visual and in-mouth/nose perception (2).

Among which, meat color, tenderness, and juiciness have been widely studied when assessing meat quality in recent decades and have been shown to be highly correlated with overall experienced quality (4). Most recent research has shown that flavor is the main driver of consumer satisfaction in lamb, but the study of flavor was limited by its complexity in the past (3). Metabolomics can measure large numbers of small molecule metabolites and help us quantify the flavor index and evaluate meat quality more comprehensively (5). Thus, combining new techniques with traditional methods to systematically evaluate sensory quality is essential to improve meat quality and meet consumer demand.

Meat quality is affected by many factors, among which intramuscular fat (IMF) content is recognized as the predominant factor (6). Previous research on mutton indicated that a certain degree of marbling is essential for optimum palatability, as IMF levels in meat are positively related to tenderness, juiciness, and flavor (7, 8). Okeudo and Moss (9) found that increasing IMF content led to decreasing shear force until the IMF content reached 3.9%. In addition, the IMF can also affect meat tenderness by alter muscle fiber characteristics (10). Although researchers have confirmed that mutton juiciness decreases with the reduction in IMF content by sensory evaluation (4, 11), few studies have substantively explored the variation in objective indices (drip loss, water holding capacity, etc.). Furthermore, a previous study indicated that flavor represents the most sensory stimulation changes that are affected by IMF content through the variation of volatile compounds (12). It is well known that flavor precursors are the basis for the formation of volatile compounds during cooking. However, the influence of IMF content on meat flavor precursors is still unclear. In total, the assessment of the influence of IMF on meat quality in lambs is not systematic enough and needs to be further explored to produce superior mutton products.

IMF deposition is impacted by multiple factors, including nutrition, feeding systems, age, animal breed, and genetic factors (13). To date, many efforts have been made to improve the IMF content in sheep through nutritional regulation or changing the feeding pattern, while these regulations are still dependent on the genetic pathway (14, 15). Mortimer et al. (16) showed that the estimated heritability of IMF content in sheep is 0.48, indicating that genetic regulation could be an effective pathway to improve the IMF content. As a typical polygenic trait, IMF deposition can be regulated by genes involved in adipogenesis and fat metabolism (17), such as *FABP*, *HSL*, *LPL*, and *DGAT1* (18–21). However, more recent studies have focused on the relationship between IMF content and fatty acid composition and found that saturated and monounsaturated fatty acids may contribute to IMF deposition (22, 23). Therefore, exploring the synthesis mechanism of fatty acids that affect IMF accumulation can prompt us to better understand the differential deposition of IMF and improve IMF content by genetic pathways in sheep.

Tan sheep are an indigenous ovine breed in China and are famous for their great eating quality and pleasant aroma (24). Due to the superior meat quality characteristics, Tan sheep has been used as a well material for sheep meat quality evaluation and research, and its meat quality traits have been widely compared with other meat sheep breeds (24, 25). However, the effect of IMF variation on meat quality in Tan sheep and the related regulation mechanism has not been systematically researched. Thus, this study was performed to explore the influence of IMF variation on meat quality from a taste and flavor perspective within Tan sheep and interpret the possible molecular mechanisms of IMF divergent deposition and flavor precursor metabolism. The current study is expected to provide a strategy for improving the mutton quality from a genetic perspective with IMF as an entry point.

## Materials and methods

### Animals and sample collection

All animal procedures performed in this study were approved by the Ethics Committee of the College of Pastoral Agriculture Science and Technology of Lanzhou University. A total of 76 Tan sheep rams with similar weights were housed in individual units (0.8 × 1.0 m^2^) and fed the total mixed ration (TMR) pellet (Supplementary Table 1) *ad libitum* from weaning to 6 months of age. Following an overnight fast of 16 h (with free access to water), the sheep were stunned, bled, and skinned according to commercial practice. After slaughter, *longissimus dorsi* tissues were collected from each carcass and divided into four parts: 30 g of the tissue was stored at −20°C for IMF content and fatty acid composition analysis; approximately 60 g of the tissue was used to assess the meat quality; approximately 1 cm^3^ tissue sample was cut into the size of a grain of rice and stored in 10% neutral formaldehyde solution for the histological analysis; and approximately 2 g was cut into small cubes frozen in liquid nitrogen and stored at −80°C until the metabolomics analysis and RNA sequencing.

### Meat quality trait assessment

The meat quality of 76 rams was analyzed on the spot. Muscle pH_45*min*_ and meat color at 45 min after slaughter were measured. Meat color was recorded three times at different locations of *longissimus dorsi* tissue using a Minolta CR-400 Chroma meter (8-mm aperture, 2° viewing angle, D65 illuminant, Konica Minolta Sensing Inc., Osaka, Japan) after 30 min of blooming at room temperature (20°), and the average values of L* (lightness), a* (redness) and b* (yellowness) were used to evaluate meat color. The pH value was measured at three different locations of the *longissimus dorsi* using a portable pH meter (HI99163, HANNA Instruments Inc., Woonsocket, RI, United States) calibrated with pH 4.0 and pH 7.0 standard buffers. The muscle pH_24*h*,_ meat color at 24 h, cooking loss, drip loss, water-holding capacity (WHC), shear force, and texture index were measured after 24 h of cooling–flushing at 4°C in a 90% humidity environment. Cooking loss and shear force values were determined as previously described with slight modifications (26). Meat samples (two replicates) were weighed and heated in a water bath at 85°C to reach a core temperature of 70°C and then cooled to room temperature (20°C). The cooked samples were wiped with filter paper to remove residual moisture and reweighed to calculate the cooking loss. Ten strips of 1 cm × 1 cm × 4 cm were cut parallel to the muscle fiber direction from the cooked samples for shear force measurement. Shear force was estimated using a C-LM3 muscle tenderness meter (Bulader Technology Development Co., Ltd., Beijing, China). Drip loss was determined as the weight loss after suspending meat samples (three replicates) in an expanded clear plastic bottle at 4°C for 24 h (27). To measure the WHC value, meat samples (three replicates) were placed between two layers of gauze with 18 layers of qualitative filter paper on the upper and lower layers. Then, the samples were pressurized to 35 kg in an RH-1000 meat pressure meter (Runhu Instrument Co., Ltd., Guangzhou, China) for 5 min, and the difference in weight was used to calculate WHC (28). A texture profile analysis (TPA) was conducted using a TMS-Pro texture analyzer (Food Technology Corporation, VA, United States).

### Intramuscular fat content measurement and fatty acid analysis

The IMF content of 76 rams was measured with the Soxhlet method (29) using petroleum ether as the solvent and quantified as the weight percentage of wet muscle tissue. According to the measurement results, twelve rams with the highest IMF content and the lowest IMF content were chosen to characterize the fatty acid profile, metabolic profile, and transcriptome profile (*n* = 6).

Fatty acids in the freeze-dried *longissimus dorsi* (0.2 g) were converted to methyl esters using acetyl chloride in methanol (30). Methyl heneicosanoate was used as an internal standard during transesterification. The fatty acid methyl esters (FAMEs) were separated on a Shimadzu GC-2014 Gas Chromatograph (Shimadzu Corporation, Tokyo, Japan) with a fused silica capillary column (HP-88, 100 m × 0.25 mm × 0.20 μm; Agilent Technologies, Co., Ltd., Santa Clara, CA, United States) and a flame ionization detector. The measurement conditions were as follows: an injection volume of 1 μL; a split ratio of 50:1; an initial temperature of 130°C for 1 min, followed by an increase to 170°C at 6.5°C/min, an increase to 215°C at 2.75°C/min, holding at 215°C for 12 min, an increase to 230°C at 4°C/min, and holding at this temperature for 3 min; and a detector temperature of 280°C. The FAMEs were identified by the FAME reference standard (37 FAME standards, Supelco, Bellefonte, PA, United States) and mixture ME 93 standard (Larodan AB, Solna, Sweden) and quantified using an internal standard calibration method.

### Histological analysis

A total of 32 rams were randomly selected from the population of 76 to conduct histological detection at Chengdu Lilai Biotechnology Co., Ltd. The methods were referenced by Cui et al. (31), with some modifications. For haematoxylin and eosin staining (HE staining), *longissimus dorsi* samples were fixed overnight in 10% neutral formaldehyde solution, embedded in paraffin, and then cut into 5 μm sections. The sections were further stained according to a standard protocol and examined with a BA210Digital light microscope (Motic China Group Co., Ltd., Xiamen, China). The fiber number in each sample was manually counted in three different fields (400 × mirror), and the average was calculated. Muscle fiber diameter and perimysium thickness were measured with Motic Images Advanced 3.2 software.

### Metabolomics analysis

For the metabolomics analysis, metabolomic profile differences were compared between rams with the highest IMF content (*n* = 6) and the lowest IMF content (*n* = 6) by LC-Bio Technology Co., Ltd. (Hangzhou, China) using widely targeted metabolomics. The metabolite was extracted according to Zhan et al. (32). In brief, samples (50 ± 2 mg of each) were homogenized at 30 Hz for 3 min with cold steel balls. Then, 1 mL of 70% methanol with internal standard extract was added to the homogenized sample in the centrifuge tube. The mixture was stirred for 5 min and then centrifuged at 12,000 rpm and 4°C for 10 min. After centrifugation, 400 μL of supernatant was placed into an EP tube and stored at −20°C overnight. After centrifugation at 12,000 r/min and 4°C for 3 min, 200 μL of the supernatant was added to an injection bottle for on-board analysis.

Metabolites were analyzed using an LC–ESI–MS/MS system (UPLC, ExionLC AD; MS, QTRAP^®^ System) with a Waters ACQUITY UPLC HSS T3 C18 (1.8 μm, 2.1 mm × 100 mm) column. To monitor and evaluate stability and reliability during detection, quality control (QC) samples were prepared by mixing all samples and inserted into the detection queue. The effluent was introduced into a high-resolution QTRAP^®^ MS/MS system, which was equipped with an electrospray ionization (ESI) Turbo Ion-Spray interface that was operated in positive and negative ion mode and controlled by Analyst 1.6.3 software (AB Sciex, Framingham, MA, United States). Instrument tuning and mass calibration were performed with 10 and 100 μmol/L polypropylene glycol solutions in QQQ and LIT modes, respectively. A specific set of multiple reaction monitoring (MRM) transitions was monitored for each period according to the metabolites eluted within this period. Based on the self-established standard substance database of Lianchuan Biotechnology Co., Ltd., the metabolites were identified according to the retention time, letter ion pair information, and secondary spectral data. Principal component analysis (PCA) and partial least squares-discriminant analysis (PLS-DA) were performed using the ropls package in R environment (version 3.6.1). The outlier sample (H_IMF_5) determined by PCA scaling plots (Supplementary Figure 1) was removed in the subsequent analysis. Differentially abundant metabolites (DAMs) were identified using the following criteria: FC (fold change) > 1.5 or FC < 0.67 and VIP (variable importance in projection) ≥ 1. The Kyoto Encyclopedia of Genes and Genomes (KEGG) pathway database was used to perform enrichment analysis of differential flavor precursors.

### Transcriptome analysis

Total RNA was extracted from *longissimus dorsi* samples of six sheep with the highest IMF content and six sheep with the lowest IMF content using TRIzol (Invitrogen, Carlsbad, CA, United States) according to the manufacturer’s instructions. The amount and purity of RNA were quantified using a NanoDrop ND-1000 UV–Vis spectrophotometer (NanoDrop, Wilmington, DE, United States). RNA integrity was assessed using a Bioanalyzer 2100 (Agilent, Santa Clara, CA, United States) with RIN > 7.0, followed by confirmation by denaturing agarose gel electrophoresis. A total of 12 RNA-seq libraries were constructed; 2 × 150 bp paired-end sequencing (PE150) was performed on an Illumina Novaseq 6000™ (LC-Bio Technology Co., Ltd., Hangzhou, China) per the manufacturer’s recommended protocol.

Raw reads with low-quality bases and contaminated with adaptors were removed to ensure that sequences were of high quality (clean reads), and Q20 and Q30 values were calculated to evaluate the quality of the clean data. The filtered sequences were aligned to the *Ovis aries* reference genome (Oar_v.3.1) using HISAT2 software. Gene expression levels were calculated as fragments per kilobase per million mapped reads (FPKM). Differentially expressed genes (DEGs) between high and low IMF content groups were identified by DESeq2 R package and filtered using the criteria: | log_2_FC| > 1 and *p* value < 0.05. The KEGG pathway enrichment analysis was performed on the DEGs using ClusterProfile package in R software (version 3.6.1). Taken *p*-value < 0.05 as a threshold, pathways that meet this condition were defined as significantly enriched pathways.

### Myosin heavy chain isoform identification and differentially expressed gene validation using quantitative real-time polymerase chain reaction

The samples and methods of RNA extraction were in accordance with those for RNA sequencing, and a total of 12 qualified RNA samples were obtained. cDNA was synthesized utilizing the TransScript One-Step gDNA Removal and cDNA Synthesis SuperMix kit (TransGen Biotech, Beijing, China) and used for myosin heavy chain (*MyHC*) isoform identification and DEG validation.

The relative expression of the *MyHC* isoforms (*MyHCI*, *MyHCIIa*, *MyHCIIx*, and *MyHCIIb*) and six randomly selected DEGs (*THRSP*, *ADIPOQ*, *FABP4*, *CIDEA*, *ACTC1*, and *PLIN1*) were determined in the *longissimus dorsi* of twelve sheep by qRT–PCR. *GAPDH* was used as the endogenous reference gene. The gene-specific primers were designed using Primer-BLAST^1^ and are shown in Supplementary Table 2. The CFX384 system (Bio–Rad, Hercules, CA, United States) was used to detect the gene expression level, and the PCR conditions were as follows: 95°C for 30 s, 40 cycles of 95°C for 5 s, and 60°C for 30 s, followed by a melt curve analysis. The relative expression levels of target genes were calculated using the 2^–ΔΔCT^ method (33).

### Statistical analysis

Differences in the IMF content, FA content, and *MyHC* isoform expression levels were determined using Student’s two-tailed *t* tests in SPSS 22.0 software (SPSS, Inc., Chicago, IL, United States); differences were considered significant when *p* < 0.05. Pearson’s correlation between DEGs and differentially abundant fatty acids as well as between IMF content and meat quality traits were determined through pairwise comparison by the Hmisc package in R version 3.6.1 software, and the correlation networks were visualized by Cytoscape version 3.9.0.

## Results

### The effect of intramuscular fat content on meat quality and muscle fiber

The average IMF content of the *longissimus dorsi* of 76 Tan sheep was 3.35 ± 0.95%, and the variation coefficient (CV) was 28.46%, indicating a large variation in IMF content within Tan sheep. Pearson’s correlation coefficient between IMF content and meat quality traits showed that IMF content had an extremely significantly positive correlation with L*, a* and b* at 45 min after slaughter (*p* < 0.01) and a significantly positive correlation with L* and a* at 24 h after slaughter (*p* < 0.05, Table 1). The results suggested that meat with low IMF content is darker than meat with high IMF content, while meat is redder and yellower in high-IMF individuals than in low-IMF individuals, and the difference in color caused by IMF variation can be alleviated by post mortem aging. However, the IMF content had no significant effect on the drip loss, cooking loss, WHC, shearing force, or texture indices.

**TABLE 1:** Pearson’s correlation of IMF content with meat quality traits and muscle fiber traits.

Traits	Mean ± SD	*r*	*p*
*Meat quality traits* (*n* = 76)			
PH_45 min_	6.73 ± 0.16	0.035	0.769
PH_24 h_	5.97 ± 0.31	0.023	0.846
Meat color (45 min)			
Lightness (L*)	35.49 ± 1.46	0.309	0.007
Redness (a*)	18.68 ± 0.77	0.323	0.004
Yellowness (b*)	2.85 ± 0.52	0.318	0.005
Meat color (24 h)			
Lightness (L*)	37.91 ± 2.34	0.238	0.038
Redness (a*)	20.93 ± 1.07	0.228	0.047
Yellowness (b*)	8.01 ± 1.42	0.168	0.148
Drip loss (%)	1.83 ± 0.74	–0.089	0.444
Cook loss (%)	39.85 ± 4.65	–0.087	0.456
Water-holding capacity (%)	26.18 ± 3.27	0.113	0.332
Shearing force (N)	82.82 ± 16.13	–0.147	0.205
Texture profile analysis			
Adhesiveness (N/mm)	0.20 ± 0.08	0.139	0.231
Cohesiveness (Ratio)	0.46 ± 0.04	0.065	0.576
Springinesss (mm)	1.79 ± 0.24	0.131	0.260
Gumminess (N)	17.25 ± 6.90	0.064	0.581
Chewiness (MJ)	32.97 ± 16.51	0.067	0.566
Hardness (N)	15.78 ± 5.38	–0.087	0.466
*Muscle fiber traits* (*n* = 32)			
Fiber Diameter (μm)	34.95 ± 3.53	0.165	0.366
Perimysium thickness (μm)	7.52 ± 2.64	0.269	0.136
Fiber number	69.74 ± 12.62	–0.461	0.008

**FIGURE 1:**
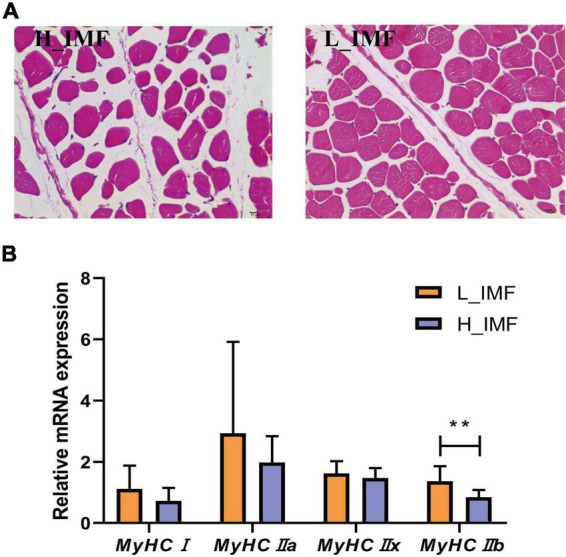
Morphological section **(A)** and muscle fiber type detection **(B)** of *longissimus dorsi*. H_IMF, high intramuscular fat group; L_IMF, low intramuscular fat group. **p < 0.01.

According to the IMF content, twelve samples were selected and allocated to two groups: a high IMF content group (H_IMF) and a low IMF content group (L_IMF). The IMF content was extremely significantly higher in the H_IMF group (5.51 ± 0.47%) than in the L_IMF group (2.03 ± 0.15%, *p* < 0.01), indicating that a lamb model with different intramuscular fat contents was constructed. HE staining demonstrated that muscle fibers were more tightly packed in the L_IMF group than in the H_IMF group (Figure 1A), corresponding to the significantly negative correlation between IMF and fiber number (Table 1). Moreover, the *MyHC* isoforms had higher expression levels in the L_IMF group than in the H_IMF group, and the expression level of *MyHCIIb* was significantly different between the two groups (*p* < 0.01, Figure 1B).

### Comparison of flavor precursor metabolites between different intramuscular fat content groups

To explore the IMF changes in response to flavor precursors, the metabolomic profile of *longissimus dorsi* was analyzed using a widely targeted metabolomics method. After removing the outlier sample, a total of 609 distinct annotated metabolites were identified in eleven samples, including 123 amino acids and their metabolites, 103 fatty acyls, 62 nucleotides and their metabolites, 78 organic compounds and their derivatives, 35 carboxylic acids and their derivatives, 63 glyceryl phosphatides, 14 oxidized lipids, and others (Supplementary Table 3).

**FIGURE 2:**
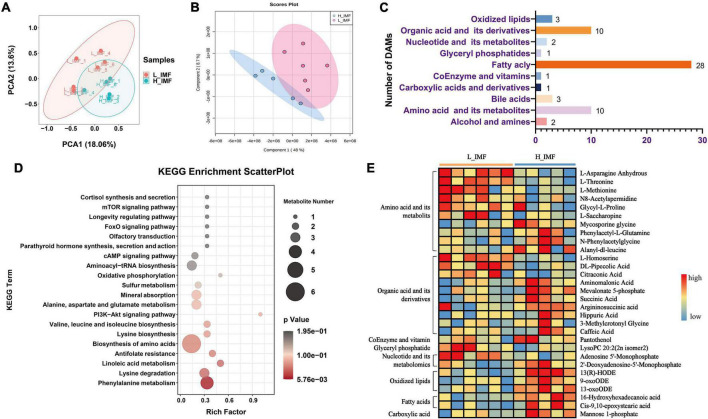
Comparison of flavor precursor profiles of Tan sheep between H_IMF and L_IMF groups. **(A)** Principal component analysis (PCA) and **(B)** partial least squares discriminant analysis (PLS-DA) score plots of the confirmed metabolites. **(C)** The number of differential abundance metabolites identified. **(D)** KEGG enrichment analysis of differential flavor precursors. **(E)** The abundance changes of differential flavor precursors between H_IMF and L_IMF groups. DAMs, differentially abundant metabolites. H_IMF, high intramuscular fat group (*n* = 5); L_IMF, low intramuscular fat group (*n* = 6).

To further differentiate the metabolomic profile between the H_IMF and L_IMF groups, the metabolomics data were analyzed using multivariate statistics. The PCA and PLS-DA score plots showed that the 609 metabolites could be further divided into two groups, indicating that there was a difference in metabolite composition between the H_IMF and L_IMF groups (Figures 2A,B). To further explore the significantly different metabolites, variables were selected on the basis of VIP values based on PLS-DA and FC values. In total, 61 metabolites were identified as DAMs between the two groups, including 28 fatty acyls, ten amino acids and their metabolites, ten organic acids and their derivatives, three oxidized lipids, three bile acids, two nucleotides and their derivatives, two alcohols and amines, one glyceryl phosphatase, one coenzyme and vitamin, and one carboxylic acid and derivative (Figure 2C). Among the 61 DAMs, fatty acyls accounted for the largest proportion, followed by amino acids and organic acids. Considering that amino acids, organic acids, vitamins, glyceryl phosphatides, nucleotides, oxidized lipids, fatty acids, and carboxylic acids contribute to flavor formation, the DAMs belonging to these categories were identified as differential flavor precursors and used for KEGG enrichment analysis. As shown in Figure 2D, multiple amino acid metabolism-related pathways were significantly enriched. The differential flavor precursor abundance analysis showed that most amino acids showed a higher abundance in the L_IMF group than in the H_IMF group. In addition, lysoPC 20:2 (2n isomer 2) and adenosine 5′-monophosphate were more abundant in the L_IMF group than in the H_IMF group, while 2′-deoxyadenosine-5′-monophosphate, mannose 1-phosphate, 13-hydroxy-9,11-octadecadienoic acid (13-HODE), 9-oxo-10 E,12 Z-octadecadienoic acid (9-oxoODE), and 13-oxo-9 Z,11 E-octadecadienoic acid (13-oxoODE) were more abundant in the H_IMF group than in the L_IMF group (Figure 2E), and they also have vital functions in meat flavor.

### Fatty acid profile variation between different intramuscular fat content groups

**FIGURE 3:**
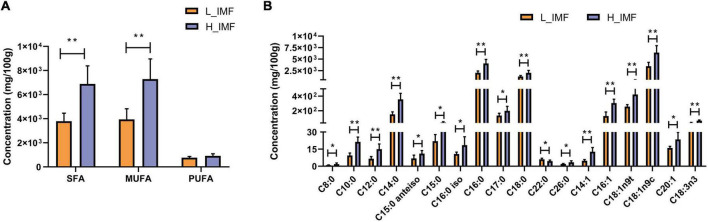
Effect of IMF content on fatty acids concentration (*n* = 6). **(A)** Concentration of total SFA, MUFA, and PUFA. **(B)** Concentration of differentially abundant fatty acids. SFA, saturated fatty acid; MUFA, monounsaturated fatty acid; PUFA, polyunsaturated fatty acid; H_IMF, high intramuscular fat group; L_IMF, low intramuscular fat group. *p < 0.05; **p < 0.01.

To explore the changes in fatty acids in lambs with the IMF content, a GC-based targeted fatty acid analysis was conducted. The fatty acid contents are shown in Figure 3. The contents of saturated fatty acids and monounsaturated fatty acids were extremely significantly higher in the H_IMF group than in the L_IMF group (*p* < 0.01), but no significant difference was observed in polyunsaturated fatty acids between the two groups (Figure 3A). Subsequently, an in-depth analysis of the variation in fatty acid molecules was performed. A total of sixteen fatty acids were significantly different, including ten saturated fatty acids, five monounsaturated fatty acids, and one polyunsaturated fatty acid. As shown in Figure 3B, the content of dodecanoic acid (C22:0) was lower in the H_IMF group than in the L_IMF group, while the contents of other fatty acids were higher in the H_IMF group than in the L_IMF group.

### Transcriptional profile analysis

**FIGURE 4:**
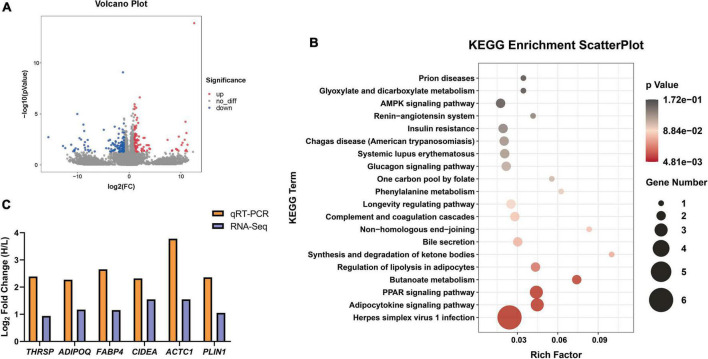
The differential expression genes (DEGs) identified between H_IMF and L_IMF groups (*n* = 6). **(A)** Volcano Plot of DEGs between two groups. **(B)** Significantly enriched KEGG pathways of DEGs. **(C)** RNA-Seq data validation.

To study the molecular regulatory mechanisms of IMF differential deposition, transcriptome sequencing was performed on twelve Tan rams with different IMF contents. In total, 77.09 Gb of clean data was obtained, and an average of 6.4 Gb of clean data for each individual with more than a 96.90% Q30 base percentage. More than 87.01% of clean reads were mapped to the *Ovis aries* genome (v.3.1), and the FPKM values between samples were highly correlated (Supplementary Figure 2, *R*^2^ > 0.85). After comparing the FPKM values between the H_IMF and L_IMF groups, 215 DEGs were identified, including 88 upregulated genes and 127 downregulated genes in the H_IMF group (Figure 4A). KEGG analysis of DEGs provided additional information about the enriched biological pathways, including lipid metabolism, carbohydrate metabolism, nucleotide metabolism, amino acid metabolism, biosynthesis of other secondary metabolites, and energy metabolism (Supplementary Figure 3). Based on the statistical significance, multiple lipid metabolism pathways were significantly enriched (*p* < 0.05), including regulation of lipolysis in adipocytes, PPAR signaling pathway, and adipocytokine signaling pathway (Figure 4B).

Six lipid metabolism-related genes (*THRSP*, *ADIPOQ*, *FABP4*, *CIDEA*, *ACTC1*, and *PLIN1*) were selected to validate the gene expression data from RNA-Seq using qRT–PCR. The expression levels of the genes were upregulated in the H_IMF group in both the RNA-Seq and qRT–PCR results (Figure 4C), indicating that the sequencing data are reliable and that IMF deposition is regulated by lipid metabolism-related genes.

### Joint analysis between metabolome, fatty acid profile, and transcriptome

**FIGURE 5:**
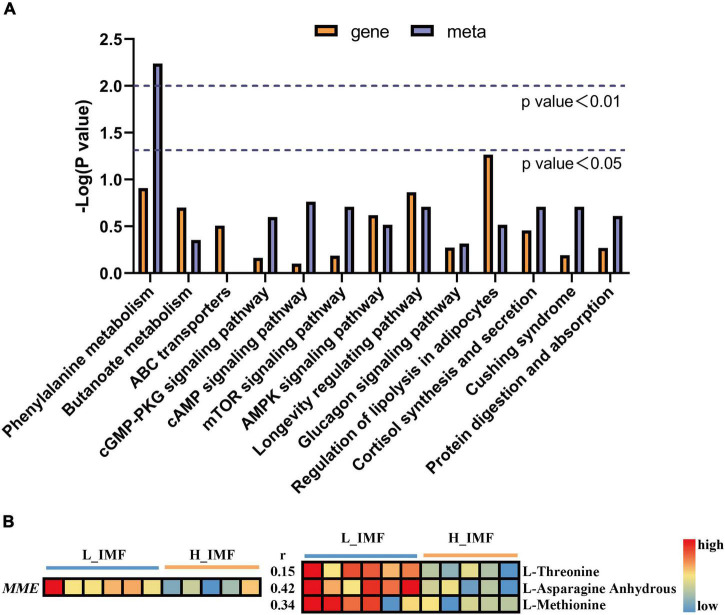
Joint analysis of transcriptome and metabolome data. **(A)** The co-enrichment pathways of transcriptome and metabolome. **(B)** The changes in *MME* gene expression level and amino acids abundance between H_IMF and L_IMF group, as well as the correlation between gene and metabolites. H_IMF, high intramuscular fat group (*n* = 5); L_IMF, low intramuscular fat group (*n* = 6).

To further explore the regulatory mechanism of DEGs on differential flavor precursor metabolism, a joint analysis of the transcriptome and metabolome data was conducted. In total, 117 and 54 pathways were enriched by DEGs and differential flavor precursors, respectively, among which thirteen pathways were shared (Figure 5A). There were ten genes and eight flavor precursors contained in shared pathways, and *MME* was found to directly regulate the metabolism of L-threonine, L-asparagine anhydrous, and L-methionine in the protein digestion and absorption pathway. Moreover, the *MME* gene expression level was downregulated in the H_IMF group, which is coincident with the abundances of L-threonine, L-asparagine anhydrous, and L-methionine (Figure 5B).

**FIGURE 6:**
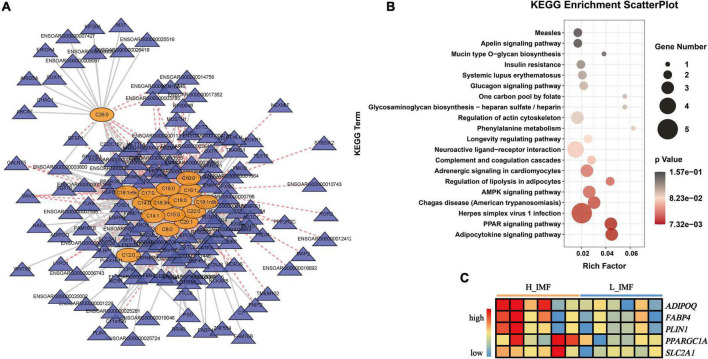
Identification of key genes regulating the metabolism of differential abundance fatty acids. **(A)** Correlation network between differential abundance fatty acids and differential expression genes (DEGs). Yellow ovals represent fatty acids; blue triangles represent DEGs; solid gray lines represent positive correlations; dashed red lines represent negative correlations. **(B)** KEGG enrichment of DEGs significantly correlated with differential abundance fatty acids. **(C)** The changes in the key gene expression level between H_IMF and L_IMF groups. H_IMF, high intramuscular fat group (*n* = 6); L_IMF, low intramuscular fat group (*n* = 6).

A joint analysis between the fatty acid profile and transcriptional profile was performed to identify the key genes regulating fatty acid metabolism and reveal the potential regulatory mechanism of IMF deposition. Pearson’s correlation analysis revealed a total of 151 DEGs significantly correlated with differentially abundant fatty acids (*p* < 0.05), and the correlation network is shown in Figure 6A. KEGG enrichment analysis found that the 151 DEGs were significantly enriched in lipid metabolism-related pathways: regulation of lipolysis in adipocytes, AMPK signaling pathway, PPAR signaling pathway, and adipocytokine signaling pathway (Figure 6B). The DEGs enriched in lipid metabolism-related pathways included *ADIPOQ*, *FABP4*, *PLIN1*, *PPARGC1A*, *SLC2A1*, and *PPP2A2C*, among which *ADIPOQ*, *FABP4*, *PLIN1*, *PPARGC1A*, and *SLC2A1* were positively correlated with saturated fatty acids and monounsaturated fatty acids and highly expressed in the H_IMF group (Figure 6C).

## Discussion

Previously, IMF deposition has been considered the most important trait influencing mutton quality, and researchers have clarified the optimal palatability threshold of IMF content [4–5%, (7, 34)] as well as the minimum acceptability [3%, (11)]. In Hu sheep, the IMF deposits rapidly at 4-05 months of age and reaches a plateau at 6 months of age, resulting in individual differences that can further affect meat quality (35). Thus, the present study detected the IMF content of the *longissimus dorsi* in Tan sheep at 6 months of age and explored the effect of IMF on meat quality. The average content of IMF in Tan sheep is 3.35% with a variation coefficient of 28.46%, indicating that the IMF content needs to be further improved to ensure an optimum eating experience. Luo et al. (36) concluded that the IMF content is weakly positively correlated with redness and yellowness in pork, and a recent study indirectly confirmed that lambs with higher IMF content have higher color traits, lightness, redness, and yellowness (37). Similarly, the IMF content of Tan sheep in this study was moderately positively correlated with meat color at 45 min and 24 h after slaughter. Meat color is primarily determined by myoglobin, which is commonly enriched in oxidized muscle fibers (type I and IIa fibers) and causes a bright meat color (38). Although all of the *MyHC* isoforms showed higher expression levels in the L_IMF group than in the H_IMF group, the relative proportion of muscle fibers may be affected by the number of total muscle fibers. Similar to Karamichou et al. (39), the number of total muscle fibers in Tan sheep showed a significant negative correlation with IMF content, which means that lambs with lower IMF content had more muscle fibers than lambs with higher IMF content. With further consideration of the lack of a significant difference in muscle fiber types except for glycolytic muscle fibers (type IIb fibers), the relative proportion of oxidized muscle fibers in H_IMF is higher, which leads to higher redness and yellowness. Meat color characteristics are considered intrinsic cues highly related to freshness and sensory quality (2), and fewer muscle fibers in lambs can effectively improve the tenderness of muscle (13). In addition, researchers have tried to use computed tomography to measure the IMF content *in vivo* (37), and the results in the present study showed that the muscle fibers are tightly packed in L_IMF lambs given the powerful evidence of the technology. The loose arrangement of muscle fiber also contributed to the higher lightness in H_IMF lambs.

Past studies have noted that IMF content is closely related to meat flavor (13, 40), and the changes in volatile aroma compounds upon IMF variation have been explored (41), while the root cause has not been clarified. The present study analyzed the effect of IMF content on mutton favor from the perspective of flavor precursors by characterizing the metabolomic profile of the *longissimus dorsi* in Tan sheep by a widely targeted metabolomics method. Frank et al. (40) reported that high muscle fat has a concentrating influence on flavor precursors and explained that with the increasing content of fat in a food matrix, molecules with hydrophilic traits will be pushed into the aqueous phase and cause the relative concentration to increase. Coincident with the above view, most of the differential flavor precursors identified in this study showed significantly higher abundance in the H_IMF group, among which mannose 1-phosphate, fatty acids, oxidized lipids, succinic acid, and nucleotides have been reported to play a vital role in meat flavor (40, 42, 43) and are an important source of the superior flavor of the meat with higher IMF content. For instance, fatty acids, and oxidized lipids can generate strong aroma compounds, such as aldehydes, unsaturated ketones, and unsaturated alcohols, all of which are the key factors determining the flavor of ruminant and non-ruminant meat (44). Nucleotides not only affect the umami flavor of the meat but also affect the aroma by degrading into carbohydrate compounds (40). Unfortunately, amino acids, as the main flavor precursors affected by IMF content variation and contributors to umami and the complex flavor of meat, were more enriched in the L_IMF group than in the H_IMF group. This phenomenon may have resulted from the increased number of muscle fibers in low-IMF meat. *MME* is a neutral endopeptidase that cleaves peptides at the amino side of hydrophobic residues (45), and the similar change tendency of *MME* gene expression levels with amino acid abundance between the H_IMF and L_IMF groups indicated that the metabolism of L-threonine, L-asparagine anhydrous, and L-methionine may be regulated by the *MME* gene. Although the detailed regulatory mechanism needs to be further verified, the results provide a genetic pathway for improving the reduction in amino acids caused by the deposition of IMF. Moreover, lipid molecules are the main ingredients of IMF, and their composition and abundance are closely associated with IMF deposition (46). While the method used in this research is more effective for detecting hydrophilic metabolites, the effect of IMF content on lipid-soluble flavor precursors in Tan sheep *longissimus dorsi* needs to be further explored using lipidomics.

In addition to being the foundation of IMF accumulation, fatty acids also play an important role in meat quality. The C18 fatty acids are essential for meat flavor. For instance, stearic acid (C18:0) contributes to the typical mutton flavor (47); oleic acid (C18:1) generates hexanal and heptanal with pleasant sweet and fatty flavors (48) as well as octanal and nonanal with fruity, fatty, and oil odors in cooked meat (49); linolenic acids (C18:3) can be cleaved to 2-pentylfuran under thermal oxidation and produce a fruity and buttery aroma (50). Thus, the higher contents of C18 fatty acids enhance the flavor of mutton with a high IMF content. Furthermore, Hodson and Linden (51) thought that fat may increase the perceived juiciness of meat by increasing parasympathetic saliva production by fatty acids. Recent studies have characterized the relationship between IMF and fatty acids and found that IMF content was linearly correlated with saturated fatty acids and monounsaturated fatty acids (22), and the saturation of lipid molecules in meat with a high IMF content is higher than that in meat with a low IMF content (52). In unpublished data from our previous research, lipid molecules with the highest correlation with IMF deposition were mainly constituted by saturated fatty acids. The results of the present study showed that the saturated fatty acids and monounsaturated fatty acids contents were significantly higher in the H_IMF group than in the L_IMF group, indicating that the regulation of IMF deposition by fatty acids is universal. A subsequent study identified five DEGs positively correlated with saturated fatty acids and monounsaturated fatty acids, among which *ADIPOQ*, *FABP4*, and *PLIN1* were the candidate genes of IMF deposition identified in a previous study (53). *FABP4* is thought to play roles in fatty acid transport as well as fat deposition by regulating lipid metabolism-related genes (54). *PLIN1* is involved in the regulation of triglyceride level and lipid droplet size in adipocytes and can maintain lipid homeostasis (55). Although *ADIPOQ* has been repeatedly identified as an IMF deposit maker, the detailed mechanism is still unclear. Furthermore, *PPARGC1A* is a coactivator of PPARG and participates in fatty acids metabolism pathways by regulating the expression of related genes (56). *SLC2A1* is a glucose transporter that is also reported to be involved in fatty acids translocation (57). Taken together, we suppose that the above genes will promote IMF deposition by regulating the synthesis of saturated fatty acids and monounsaturated fatty acids and then improve meat quality in Tan sheep. The detailed regulation mechanism needs to be further explored in future research.

## Conclusion

In summary, the present study is the first to systematically investigate the effect of IMF differential deposition on the meat quality of Tan sheep as well as to explore the genetic mechanism of flavor precursor metabolism and IMF accumulation. The results show that the IMF content was significantly positively correlated with the meat color at 45 min and 24 h after slaughter and affected the amount and type of muscle fiber. The saturated fatty acids and monounsaturated fatty acids contents were significantly higher in lambs with a high IMF content than in those with a low IMF content. Furthermore, a total of 61 DAMs were identified between different IMF content groups, among which amino acids are the main differential flavor precursors, and the *MME* gene can directly regulate the metabolism of amino acids. The joint analysis between the fatty acid profile and transcript profile revealed that *ADIPOQ*, *FABP4*, *PLIN1*, *PPARGC1A*, and *SLC2A1* promote IMF accumulation by positively regulating saturated fatty acids and monounsaturated fatty acids synthesis metabolism. These findings promote our understanding of IMF affecting meat quality in Tan sheep and would help improve the IMF content by genetic pathways.

## Data availability statement

The data presented in this study are de posited in the NCBI SRA, accession number PRJNA794212.

## Ethics statement

The animal study was reviewed and approved by Ethics Committee of the College of Pastoral Agriculture Science and Technology of Lanzhou University.

## Author contributions

XY and XZ: conceptualization. XZ, CL, and YK: investigation. XZ: visualization. XZ, XY, and FL: writing—original draft. XZ and XY: writing—review and editing. All authors have read and agreed to the published version of the manuscript.

## Footnotes

1
http://www.ncbi.nlm.nih.gov/tools/primer-blast/
