# Supplementary material for: Effects of intramuscular fat on meat quality and its regulation mechanism in Tan sheep
Source: Front Nutr. 2022 Jul 28;9:908355. doi: 10.3389/fnut.2022.908355 (PMC9366309; doi:10.3389/fnut.2022.908355)

Supplementary Figure S1 The outlier sample determined by PCA scaling plots.

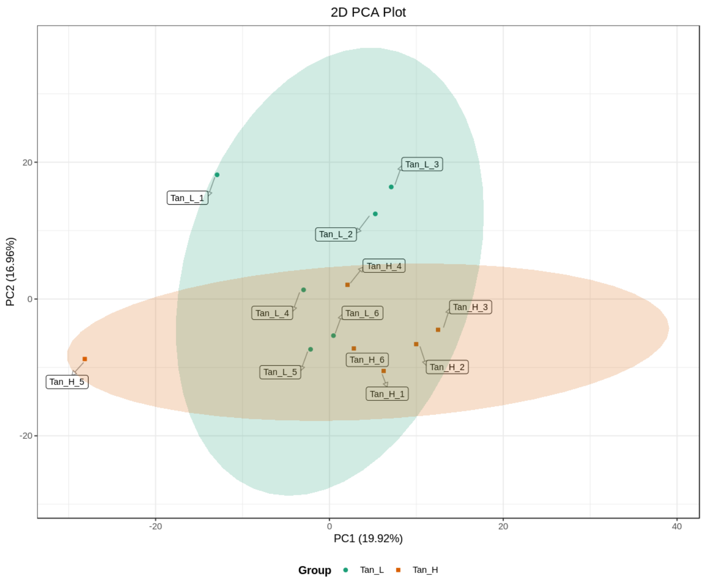

Supplementary Figure S2 Pearson correlation between samples used in RNA-Seq.

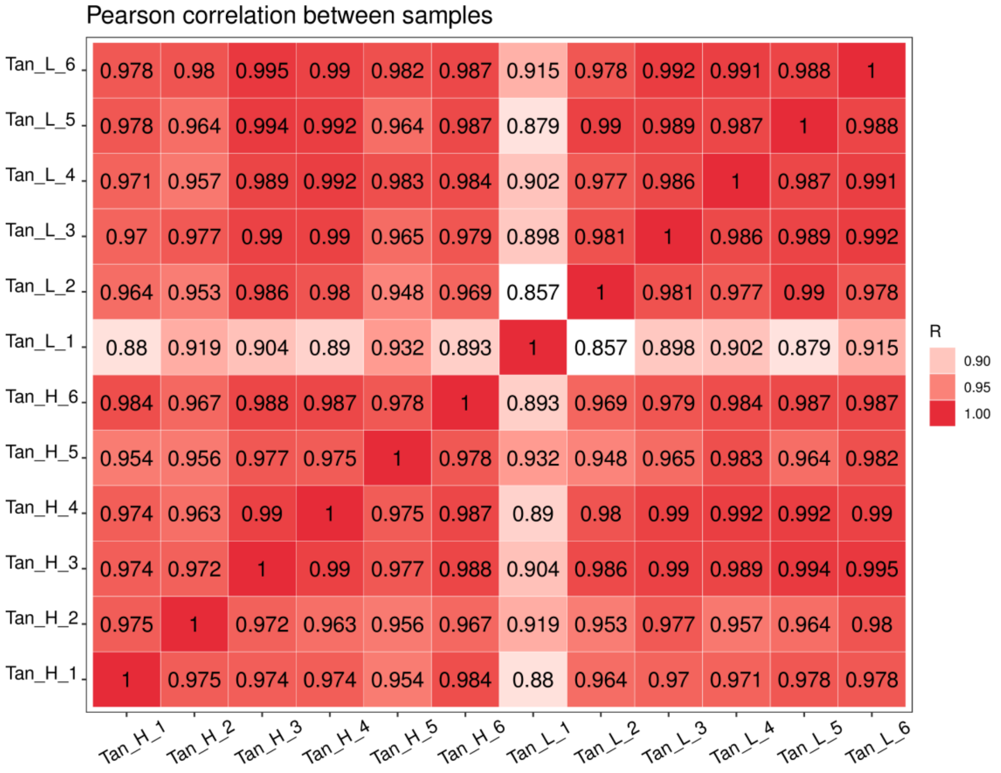

Supplementary Figure S3 KEGG enrichment analysis of differentially expressed genes.

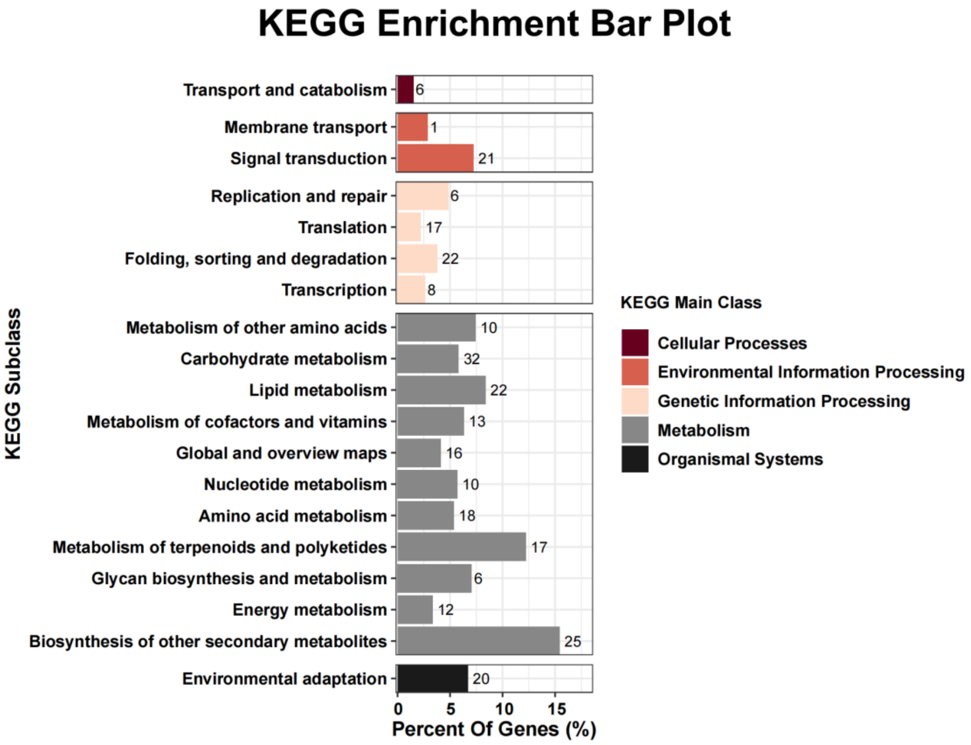

Supplement: Supplementary file 1 [file Presentation_1.pdf]
