# Supplementary material for: Effects of intramuscular fat on meat quality and its regulation mechanism in Tan sheep
Source: Front Nutr. 2022 Jul 28;9:908355. doi: 10.3389/fnut.2022.908355 (PMC9366309; doi:10.3389/fnut.2022.908355)
Supplement: Supplementary file 2 [file Data_Sheet_1.docx]

**Supplementary Table S1** Ingredients and chemical compositions of the TMR diets.

| Items |  |
| --- | --- |
| Ingredient composition (% as fed) |  |
| Corn | 33.2 |
| Corn germ meal | 18 |
| Corn stalks | 12 |
| Corn hulls | 11.2 |
| Corn cob | 8 |
| Soybean meal | 5 |
| Cotton meal | 5 |
| Molasses | 3.3 |
| Bentonite | 1.5 |
| Baking soda | 1 |
| Stone powder | 0.8 |
| Expanded Urea | 0.5 |
| Premix | 0.5 |
| Chemical composition (%) |  |
| Dry matter | 88.78 |
| Crude protein | 13.09 |
| Digestible energy | 11.11 |
| Neutral detergent fibre | 27.08 |
| Acid detergent fiber | 13.99 |
| Crude fiber | 9.78 |

**Supplementary Table S2** Primer sequences of qRT-PCR

| gene/ACC. # | Sequence (5' to 3') | Size  (bp) |
| --- | --- | --- |
| *FABP4* | F: AAGAAGTGGGTGTGGGCTTT | 91 |
| *EU301804* | R: ATGTTGACCACATCCCCATT |  |
| *PLIN1* | F: GATCGCCTCTGAGCTGAAGG | 108 |
| *XM_015101761.2* | R: AGAGCGGCCCCTAGGATTT |  |
| *THRSP* | F: TCTTACCCTGAAAGCCGAGG | 119 |
| *XM_004019431* | R: TGTGGAGTAAGCTTTCCTACATGG |  |
| *CIDEA* | F: TTGGTGCTGGAGGAGGATGG | 272 |
| [*XM_042239293.1*](https://www.ncbi.nlm.nih.gov/entrez/viewer.fcgi?db=nucleotide&id=2062854552) | R: CCGGTGCAGTGGATGTCGTA |  |
| *ADIPOQ* | F: ATCCCCGGGCTGTACTACTT | 138 |
| *NM_001308565* | R: CTGGTCCACGTTCTGGTTCT |  |
| *ACTC1* | F: ATTATTGCTCCCCCTGAGCG | 231 |
| *XM_004010972* | R: TGAGAGATGAGGGAGGGTGG |  |
| *GAPDH* | F: CACCCTCAAGATTGTCAGC | 114 |
| *NM_001190390* | R: CAGTGGTCATAAGTCCCTCC |  |
| *MyHC* I | F: GCAAGAAGAGGAGTGAGGCA | 170 |
| *XM_004010325.3* | R: GGCAGCAATGACCGCAAA |  |
| *MyHC* IIa | F: CTGAGGAGGCTGAGGAACA | 175 |
| *XM_027974884.1* | R: TCAGGACACGATCACTCTTCA |  |
| *MyHC* IIx | F: AACAACTTCCAGAAACCCAAAC | 147 |
| *XM_027974882.1* | R: GTACAGCCCGACCACCGT |  |
| *MyHC* IIb | F: TGAGGCAACAAAGAATCTTAGAAAC | 60 |
| *XM_027974883.1* | R: AAGTGGAGCTGAGTGTCCTTC |  |
